# Blue nevus of the prostate

**DOI:** 10.4103/0970-1591.65411

**Published:** 2010

**Authors:** Ranjini Kudva, Padmaraj Hegde

**Affiliations:** Department of Pathology, Kasturba Medical College, Manipal, India; 1 Urology, Kasturba Medical College, Manipal, India

A 53-year-old man presented with history of dysuria. Clinical and radiological findings were compatible with benign prostatic hyperplasia. Serum PSA level was normal. Transurethral resection of prostate was performed. The specimen weighed 8 g and on histopathological examination disclosed glandular-stromal hyperplasia and extensive black pigmentation of the stroma. Stroma showed polygonal, elongated and fusiform cells with abundant brownish black granular pigment in the cytoplasm, obscuring the nucleus. The glandular epithelial cells were free of the pigment[Figure 1]. The pigment was negative for iron with the Perls’ Prussian blue stain. The Fontana Masson silver impregnation for melanin was strongly positive. The pigment was bleached after treatment with potassium permanganate. These results suggest that the pigment in the stromal cells was melanin. The pigmented stromal cells stained with S-100 protein[Figure 2] and hence a diagnosis of blue nevus was rendered. The patient on follow-up after nine months was free of any disease.

**Figure 1:**
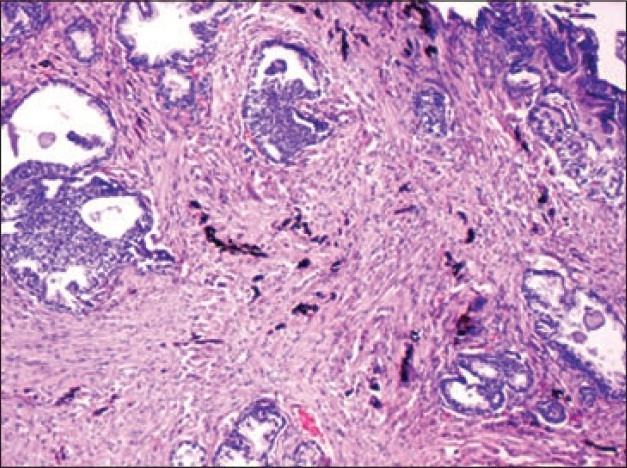
Polygonal and elongated cells with black pigment in the prostatic stroma (H and E, ×100)

**Figure 2:**
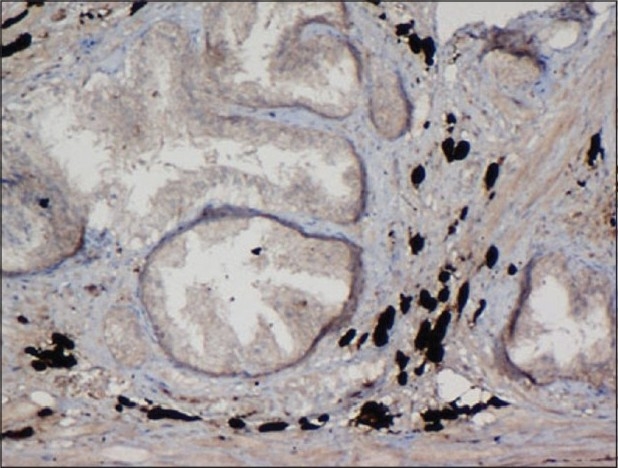
The pigmented stromal cells show immunopositivity for S 100 protein, ×200

## DISCUSSION

Blue nevus is a rare lesion of dermal melanocytes. Although it usually occurs in the skin, it has been reported in other locations like oral mucosa, sclera, cervix, vagina and prostate. Blue nevus of the prostate is a rare lesion of dendritic melanocytes which may be found in the fibromuscular stroma. Melanin in the prostate was first documented by Nigogosyan and coworkers in 1963.[1] It was encountered in ovoid and elongated melanocytes in the fibromuscular stroma and designated blue nevus of the prostate owing to its similarity to the common blue nevus of prostate.

Melanocytic lesions of the prostate are exceedingly rare and may be found in three different forms— melanosis, blue nevus and malignant melanoma either primary or metastatic. Melanosis is a condition in which melanin is found in the stromal cells of the prostate and the glandular epithelium.[2] Melanosis has been reported in 0.07–10% of normal prostatic glands.[3] Blue nevi of the prostate are similar to those in the skin and are characterized by elongated fusiform melanin-containing cells in the stroma.[2] We have reviewed the literature and summarized the salient features in Table 1. It is important to remember that blue nevus can occur in the prostate so as not to misdiagnose them as malignant melanoma. Primary malignant melanoma is described in the prostate.[4] It is important that these lesions are carefully distinguished from cellular blue nevi to avoid over-diagnosis of malignancy.

**Table 1:** Review of reported cases of blue nevus of prostate

Authors	Age in years	Symptoms	Pigment visible grossly	Pigment in stromal cells	Pigment in both epith and stromal cells	Follow up
R Martinez *et al*.	58	Lower UT obstruction	-	+	-	NA
Martinez *et al*.	80	“	-	+	-	NA
Ro *et al*.	1-68	“	+	+	-	1-died of
( 2 cases)	2-76					unrelated cause 2-Lost for follow up after 1 yr
Kovi *et al*.	65	“	-	+	-	NA
Langley *et al*.	NA	“	-	+	-	NA
Nigogosyan *et al*.	50	Post mortem( c/o M.myeloma on treatment)	+	+		Post mortem
Present case	53	Lower U T obstruction	-	+	-	No symptoms

UT= Urinary tract, NA = Not available

The origin of melanocytes within the fibromuscular stroma of the prostate remains speculative. Rawles demonstrated that melanoblasts arise from the neural crest and migrate through the body until they reach their ultimate tissue site where they differentiate into recognizable melanocytes and the melanin is produced by melanocytes in the stroma.[2] Melanin in the glandular epithelium of the prostate as is seen in melanosis is thought to be the result of transfer of pigment from the adjacent stromal melanocytes into the glandular epithelium.[5,6] Pigment which can be seen in prostatic epithelial cells unassociated with melanocytes has features of both melanin and lipofuchsin and hence represents an endogenous lipofuchsin-like cellular product rather than an example of melanogenesis by prostatic epithelial cells.[6]

It is clinically important to recognize blue nevus of prostate because this condition may closely simulate a malignant melanoma involving the gland. Blue nevus of the prostate may be overlooked because most often it is composed of small microscopic foci of relatively few pigmented cells in the fibromuscular stroma.[6]
